# Superhydrophobic bilayer coating for passive daytime radiative cooling

**DOI:** 10.1515/nanoph-2023-0511

**Published:** 2023-10-18

**Authors:** Bin Zhao, Chengfeng Xu, Cheng Jin, Kegui Lu, Ken Chen, Xiansheng Li, Lanxin Li, Gang Pei

**Affiliations:** Department of Thermal Science and Energy Engineering, University of Science and Technology of China, Hefei 230027, China

**Keywords:** radiative cooling, superhydrophobicity, synergetic reflection, atmospheric window

## Abstract

Passive radiative cooling is an energy-free cooling method by exchanging thermal radiation with the cold universe through the transparent atmospheric window. Spectrum tailoring of the radiative cooler is the key to daytime radiative cooling in previously reported works. In addition, radiative coolers with large-scale fabrication and self-cleaning characteristics should be further developed to improve their industrial applicability. Herein, we propose a bilayer radiative cooling coating with the superhydrophobic property and a scalable process, by covering TiO_2_/acrylic resin paint with a silica/poly(vinylidene fluoride-co-hexafluoropropylene) (SiO_2_/P(VdF-HFP)) composite masking layer. The strong Mie scattering in TiO_2_/acrylic resin paint contributes to high solar reflection, while the SiO_2_/P(VdF-HFP) masking layer is responsible for superhydrophobicity and synergetic solar reflection in the ultraviolet band, resulting in an effective solar reflectivity of 94.0 % with an average emissivity of 97.1 % and superhydrophobicity with a water contact angle of 158.9°. Moreover, the as-fabricated coating can be cooled to nearly 5.8 °C below the temperature of commercial white paint and 2.7 °C below the local ambient temperature under average solar irradiance of over 700 W m^−2^. In addition, yearly energy saving of 29.0 %–55.9 % can be achieved after the coating is applied to buildings in Phoenix, Hong Kong, Singapore, Guangzhou, and Riyadh.

## 1 Introduction

Radiative cooling is a new and passive cooling method with zero energy consumption, which is pollution-free and emission-free compared to traditional cooling methods, such as compression-type refrigerating. Based on the transparency of atmospheric window (8–13 μm), objects on Earth can continuously exchange thermal radiation with the cold universe, resulting in a net radiative energy flow toward space and a cooling effect on the object’s surface [1–6]. At present, the climate change problem is serious, and clean cooling techniques are emergently required. Fortunately, passive radiative cooling coincides precisely with the cooling demand throughout the whole day, particularly during the daytime, which can provide continuous cooling effects passively for various applications, such as buildings [7–9], photovoltaic cooling [10–12], power generation [13–15], color surface cooling [16], and textile cooling [17–19], contributing to significant energy-saving benefits.

Over the last decade, radiative cooling has been achieved through different kinds of radiative coolers with strong solar reflection and high long-wave emissivity, including multilayer photonic material [20–23], polymer-based coatings [24–26], porous structure [27–30], and metamaterials [4, 31]. Although the spectrum property of the radiative coolers is optimized to an excellent level, the practical application and large-scale production of these materials are still limited because of the complex fabrication process and expensive manufacturing costs. For example, multilayer photonic materials are usually fabricated by sophisticated deposition processes and metal-polymer coatings generally require precious metals (e.g., Ag) as a back solar reflective film. Polymer-based materials also face similar problems due to expensive components or complicated production conditions, such as electrospinning or high temperatures.

Recently, radiative cooling paint has been proposed as a scalable and effective radiative cooler [32–38]. Paint composed of particles and matrix has the advantages of low cost, simple manufacturing, and friendly for the user, which is a potential candidate to achieve efficient radiative cooling in industrial cases. Paint doped by particles with large electronic band gaps, such as calcium carbonate (CaCO_3_) [39], barium sulfate (BaSO_4_) [34], and hexagonal boron nitride (hBN) [40], can achieve effective full-band solar reflection, but the high particle concentration is recommended. BaSO_4_-acrylic paint with a volume concentration of 60 % can reflect over 98 % solar irradiance and obtain an average net cooling power of over 100 W m^−2^ [34]. However, a high volume concentration of particles in the paint will cause real-world issues, including cost and reliability (e.g., cracking). Generally, it’s widely believed that particles with higher refractive index differences with the matrix can scatter the sunlight more effectively [41]. Compared with the above article, titanium dioxide (TiO_2_) has a high refractive index of 2.7, so it has the potential to achieve strong solar reflection in the matrix even with low concentrations. Besides, the TiO_2_ particle serves as a kind of most common particle in industrial white paint with a low cost, a good feature for industrial use. However, TiO_2_ has a relatively low bandgap of 3.0 eV, resulting in a sharp absorption of the ultraviolet (3.2 eV) and violet light (∼7 % of solar energy) [33], which causes an additional solar heating effect under sunlight [42], as well as suffering from the ultraviolet aging problem. Therefore, improving the ultraviolet reflection for TiO_2_-based paint is an attractive way to develop cost-friendly radiative cooling paints, and this is what we do in this work.

It is worth noting that radiative coolers are usually exposed outdoors all the time in industrial cases (e.g., rooftop application), which inevitably face contaminations from the environment. The dust flowing in the air will accumulate and deposit on the radiative coolers during the long operation period, which will deteriorate the original spectral property of the cooler and then reduce radiative cooling performance, as well as lifetime. Importantly, coolers like paints are normally hydrophilic in wetting properties [43] and the water drops from rain or dew can easily adhere to the surface and even penetrate the cooler, which may irreversibly destroy the cooler structure. Therefore, it is crucial to integrate the self-cleaning property into the existing radiative cooling paints [44–47]. Importantly, the potential conflicts between the optical and superhydrophobic properties of the radiative cooling paints should be highly avoided. Furthermore, the requirement for low-cost and large-scale manufacturing also introduces an additional challenge.

In this work, a superhydrophobic bilayer radiative cooling (SBRC) coating with the spectrum tailoring property and a scalable process is proposed and fabricated by spraying a SiO_2_/P(VdF-HFP) composite masking layer to a TiO_2_/acrylic resin paint. The strong Mie scattering in particle size–optimized TiO_2_/acrylic resin paint contributes to high solar reflection. Besides, the SiO_2_/P(VdF-HFP) composite masking layer not only possesses superhydrophobic properties but also synergetically enhances the light reflection in the ultraviolet band where TiO_2_ exhibits intrinsic absorption. Besides, all the acrylic resin, SiO_2_, and P(VdF-HFP) are thermally emissive, corresponding to a high long-wave emissivity. Consequently, the SBRC coating achieves an AM1.5 spectrum-weighted solar reflectivity of 94.0 % and simultaneously exhibits a strong broadband emission with an average emissivity of 97.1 %, a good feature for daytime radiative cooling. Moreover, the SBRC coating possesses superhydrophobic properties with a water contact angle of 158.9°, showing a self-cleaning function. Outdoor testing further demonstrates that the SBRC coating can be passively cooled to be nearly 5.8 °C lower than that of commercial white paint and 2.7 °C below the local ambient temperature under direct sunlight, exhibiting potential for industrial applications. Building simulation reveals that yearly energy saving of 29.0 %–55.9 % can be achieved by SBRC coating integrated buildings in Phoenix, Hong Kong, Singapore, Guangzhou, and Riyadh.

## 2 Materials and methods

### 2.1 Materials

Acrylic resin (BA-201) is purchased from Shandong Yousuo Chemical Technology Co., Ltd (China). TiO_2_ particles (diameter: 500 nm) and SiO_2_ particles (diameter: 500 nm) are acquired from Yumu New Materials Co., Ltd (China). Additives are purchased from Nanjing Chuangshi Chemical Additives Co., Ltd (China). P(VdF-HFP) and solvents (e.g., ethyl alcohol and acetone) are purchased from the Drug Store of the University of Science and Technology of China. 1*H*,1*H*,2*H*,2*H*-Perfluorooctyl trichlorosilane (PFOCTS) is purchased from Macklin Biochemical Technology Co., Ltd (China). Commercial radiative cooling paint is purchased from Chongqing Lanyezi New Material Co., Ltd (China).

### 2.2 Preparation of the SBRC coating

Acrylic resin and TiO_2_ particles are mixed with a weight ratio of 10:1, and the mixtures are then stirred at room temperature for more than 5 h to obtain the uniform white slurry solution. During this process, additives, including coupling agent, film-forming agent, and leveling agent, are added to the mixture. The prepared white slurry solution is scraped onto the substrate and then naturally solidified for one day to form the TiO_2_/resin paint layer.

For masking layer preparation, a solvent mixture (ethyl alcohol/acetone) is first prepared by mixing 8.0 g ethyl alcohol and 5.5 g acetone, respectively. Then, 0.8 g SiO_2_ particles and 1 g P(VdF-HFP) are added to the previous solvent mixture and a stirring operation is required to obtain the SiO_2_ suspension. Amount of 70 μL PFOCTS is then added to the suspension for hydrophobic modification. During the above mixing and hydrophobic modification process, a stirring process is maintained for more than 6 h with an ultrasonic dispersion process for 0.5 h to ensure uniform mixing and complete dispersion. Next, the mixed suspension is sprayed onto the front surface of the TiO_2_/resin paint layer to form the masking layer, and the sample is then placed in a ventilated space for a natural dry process until the acetone and ethyl alcohol are completely evaporated. Finally, the SBRC coating is successfully prepared.

### 2.3 Materials characterizations

The spectral reflectivity (*R*(*λ*)) of the SBRC coating in the solar radiation band (i.e., 0.3–2.5 μm) is measured using a UV–Vis–NIR spectrophotometer (SolidSpec-3700 DUV, Shimadzu) equipped with a Teflon-coated integrating sphere. The AM 1.5 weighted average solar reflectivity (*R*) is then calculated by:

(1) $$R=\frac{\overset{2.5}{\underset{0.3}{\int }}R\left(\lambda \right)I_{\text{AM}1.5}\left(\lambda \right)\text{d}\lambda }{\overset{2.5}{\underset{0.3}{\int }}I_{\text{AM}1.5}\left(\lambda \right)\text{d}\lambda }$$

The long-wave reflectivity of the SBRC coating is measured by Fourier transform infrared (FT-IR) spectrometer (Bruker VERTEX 80) equipped with a gold-coated integrating sphere from 6 μm to 20 μm. Then, the long-wave emissivity (*ε*(*λ*)) of the SBRC coating is determined by the energy balance and Kirchhoff’s law. The average emissivity (*ε*) can be weighted by the blackbody thermal radiation:

(2) $$\varepsilon =\frac{\overset{20}{\underset{6}{\int }}\varepsilon \left(\lambda \right)I_{\text{bb}}\left(\lambda ,T\right)\text{d}\lambda }{\overset{20}{\underset{6}{\int }}I_{\text{bb}}\left(\lambda ,T\right)\text{d}\lambda }$$

The surface and cross-sectional morphology is observed by field emission scanning electron microscopy (ZEISS Gemini SEM 500). The distribution of chemical elements of SBRC coating is detected by an energy dispersive spectrometer attached to SEM. The superhydrophobicity of the SBRC coating is characterized by water contact angles (CAs) and sliding angles (SAs), which are measured using a contact angle goniometer (Ramé-hart 290-U1, USA) with the help of a water droplet of 10 μL.

### 2.4 Outdoor experiment testing

Outdoor experiment tests of radiative cooling performance were conducted on sunny days in March 2023 at the rooftop of the second Mechanics building at the University of Science and Technology of China in Hefei, China (32°N, 117°E). The experimental setup is divided into two catalogs that include a closed system and an open system. For the closed system, identical polystyrene (PS) foam boxes (25 cm × 25 cm × 9 cm) equipped with enclosing cavities (6.5 cm × 6.5 cm × 3 cm) are manufactured. All the surfaces of the above boxes, including the inner surfaces of the chamber, are covered by highly reflective aluminum foil to minimize the parasitic solar heating effect. SBRC coating and reference samples are fixed in the cavities with a transparent polyethylene (PE) cover. T-Type thermocouples are attached to the backside of the samples to monitor the temperature of samples. Besides, T-type thermocouples are also fixed in the cavity behind the sample to measure the local ambient temperature. Notably, we define the temperature of the air at a shaded region inside the cavity as the local ambient temperature in this work. For the open system, SBRC coating and reference samples are attached to a flat polystyrene (PS) foam and directly exposed to the surroundings.

During the testing, ambient temperature and relative humidity are measured by a weather station (HSTL-BYXWS), which contains a thermometer shelter according to the guidance of meteorological science. Wind speed is measured by a three-cup anemometer (NHFSXY2808), and solar irradiance is measured by a pyranometer (TBQ-2, Jinzhou Solargiga Energy Co., Ltd). All of the above-mentioned sensors are connected to a data logger (HIOKI LR8450) to record the data.

### 2.5 Building energy-saving simulation

Building energy-saving simulations are performed using EnergyPlus. A self-designed building with a size of 8.0 m × 6.0 m × 2.7 m is applied as the reference building. The information on the envelope structures and their thermophysical properties is presented in Table S1 (see Supplementary Material). For the HVAC system, an ideal load air system is applied, which supplies cooling or heating air to a zone in sufficient quantity to meet the zone load. The baseline set point for the indoor air temperature is 24 °C in summer and 20 °C in winter. The SBRC coating is assumed like a massless layer with the same optical properties as the above fabricated SBRC coating and is covered on the exterior wall of the building. Five cities, including Phoenix, Hong Kong, Singapore, Guangzhou, and Riyadh, are selected for energy-saving simulation, and the typical meteorological year data of the above cities [48] are input as the boundary for energy-saving simulation.

## 3 Results and discussion

### 3.1 Fabrication and superhydrophobic characterizations of the SBRC coating

**Figure 1:**
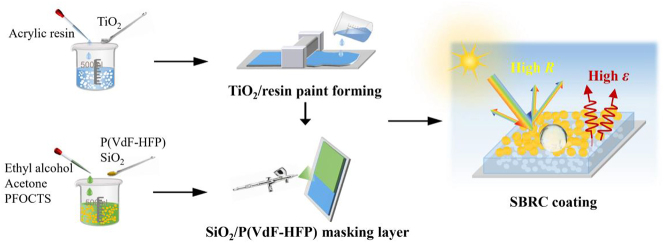
Schematic illustration of the fabrication process of SBRC coating.

The SBRC coating is fabricated based on a two-step process (Figure 1), involving the preparation of the TiO_2_/resin paint and the introduction of the masking layer. Firstly, the TiO_2_/resin paint is prepared by mixing acrylic resin and TiO_2_ particles, followed by thorough electromagnetic stirring and film formation via a blade-coating method. Then, the masking layer is prepared by spraying hydrophobically modified SiO_2_/P(VdF-HFP) mixture to the surface of solidified TiO_2_/resin paint to form the SBRC coating. The TiO_2_/resin paint and masking layer are designed to synergistically contribute to the full-band strong solar reflection, while the top masking layer is responsible for the superhydrophobic and self-cleaning properties. Notably, the above fabrication solution does not require complex processes or equipment, implying that it could be extended to achieve large-scale manufacturing for industrial cases.

**Figure 2:**
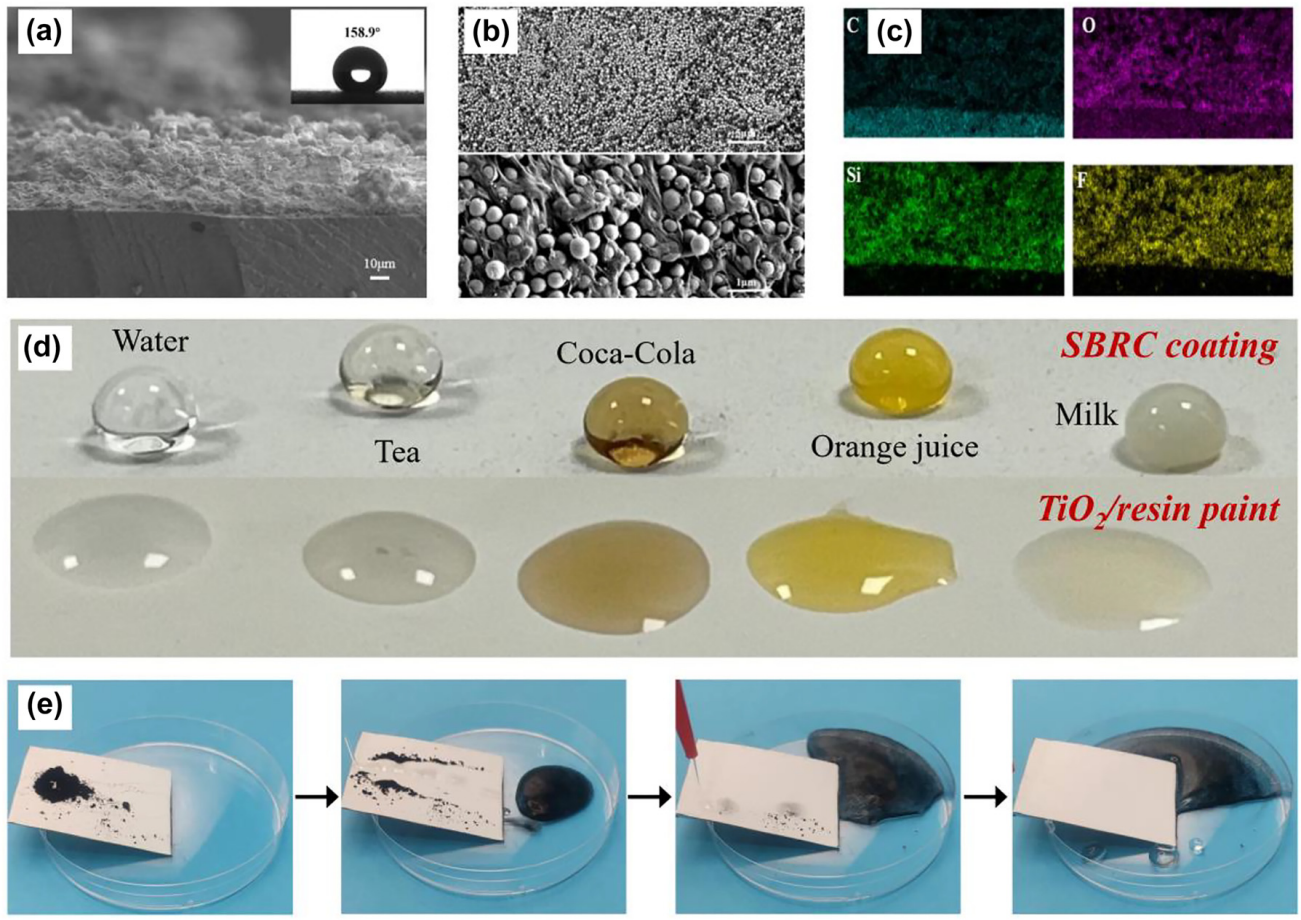
Morphology and superhydrophobic characterizations of the SBRC coating. (a) and (b) Cross-sectional view and top-view SEM images. (c) Element mapping of C, O, Si, and F of the cross-sectional view. (d) Photo of the SBRC coating with various droplets, including deionized water, tea, Coca-Cola, orange juice, and milk. (e) Self-cleaning characterization process. The carbon nanotubes are easily removed by water droplets.

The masking layer is a SiO_2_/P(VdF-HFP) composite layer, which behaves as a nonperiodic micro–nano structure on the top surface of the SBRC coating due to the clustering and gradual accumulation of SiO_2_/P(VdF-HFP) mixture during the spraying process (Figure 2a). In addition, the SiO_2_/P(VdF-HFP) composite layer exhibits a textured structure with protruding SiO_2_ nanoparticles and interparticle gaps [43] (Figure 2b), which dramatically reduces the contact phenomenon between water droplets and the SBRC surface, resulting in the superhydrophobicity of the SBRC coating. The enlarged top-view SEM image (Figure 2b) also shows that a “Chain-Bead” structure [49] is formed by randomly combining P(VdF-HFP) and SiO_2_ particles, where chain-like fibers connect the SiO_2_ nanoparticles to strengthen the bonding [50]. In addition, the element distribution of C, Si, O, and F (Figure 2c) indicates the uniform distribution of SiO_2_ particles and P(VdF-HFP) during the spraying process.

The SBRC coating shows surface superhydrophobic properties with a contact angle of 158.9° (Figure 2a), and such a superhydrophobicity possesses excellent liquid-repellent function for different droplets, including deionized water, tea, Coca-Cola, orange juice, and milk (Figure 2d), with droplets on the coating surface contracting into nearly spherical shapes. By contrast, the TiO_2_/resin paint exhibits hydrophilia, and the above droplets behave like flat sheets. Importantly, the superhydrophobic properties provide a unique function of self-cleaning for the SBRC coatings, and it can be seen from Figure 2e that the droplet will become spherical and quickly roll off, carrying away the dust in their rolling path, until the surface is restored to a clean state, which can prevent the coating from dust, sewage, and other pollutes, always making the coating works in a good state (Movies S1 and S2).

### 3.2 Optical characterizations of the SBRC coating

**Figure 3:**
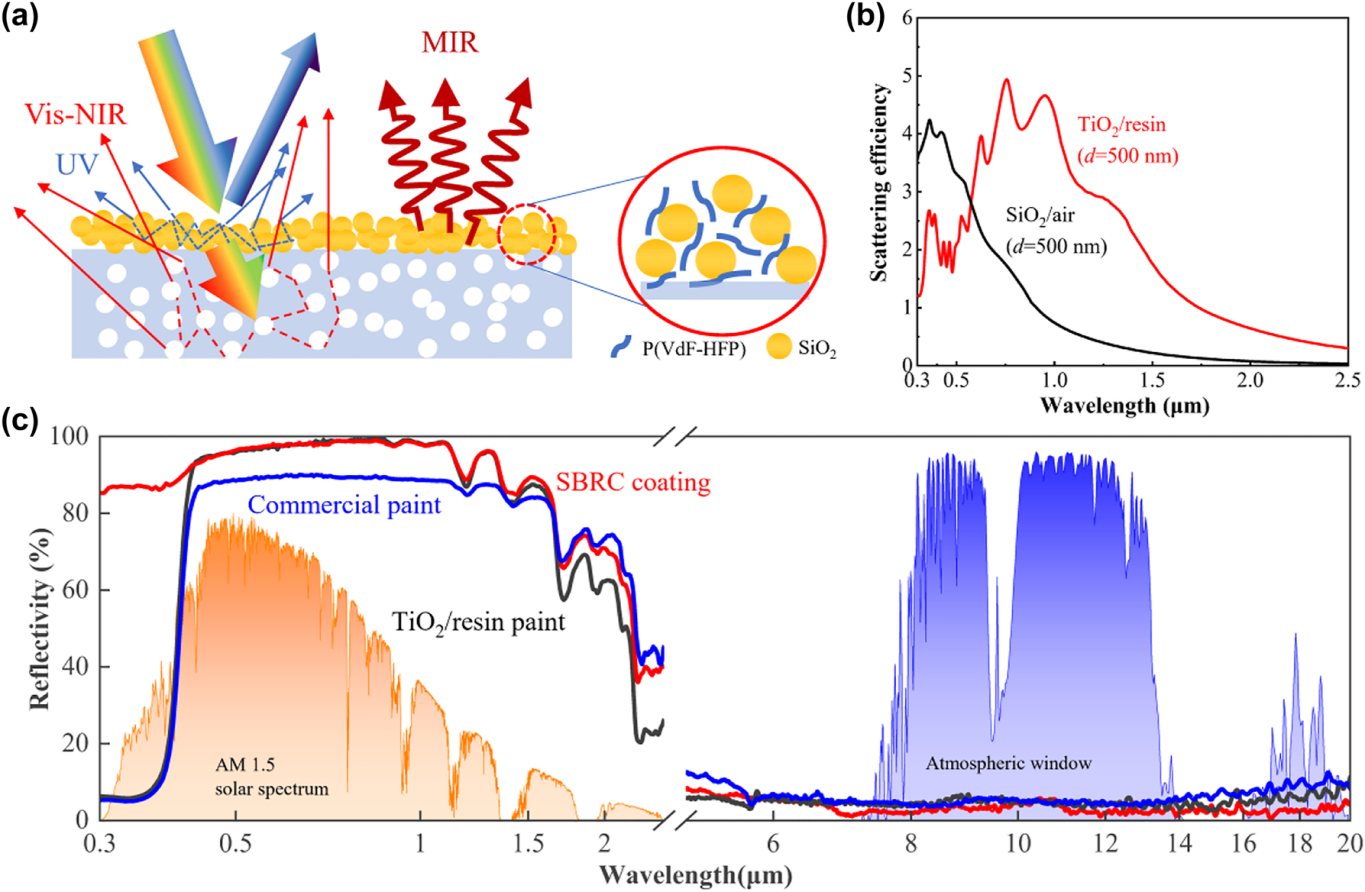
Optical properties of the SBRC coating. (a) Schematic of the scattering and thermal emission principle. The bottom TiO_2_/resin paint contributes to solar reflection in the visible and near-infrared bands, while the top masking layer is responsible for the synergistic solar reflection, especially in the ultraviolet band. Thermal emission is contributed by the combined emission of SiO_2_, P(VdF-HFP), and acrylic resin. (b) Scattering efficiency of TiO_2_ and SiO_2_ particles with an optimized size of 500 nm, the reflection of the bottom and top layer of the SBRC coating can synergistically reflect incident light. (c) Spectral reflectivity of the SBRC coating, TiO_2_/resin paint, and commercial paint from ultraviolet to the mid-infrared band, with AM1.5 solar spectrum and transmittance of atmospheric window plotted as references.

SBRC coating consists of two layers, including TiO_2_/resin paint and SiO_2_/P(VdF-HFP) composite masking layer (Figure 3a). The TiO_2_/resin paint with TiO_2_ particles randomly dispersed in the resin matrix has a strong scattering effect on sunlight, corresponding to a high solar reflection. However, the intrinsic absorption of TiO_2_ causes a sharp absorption in the ultraviolet band, which limits the solar reflection of TiO_2_/resin paint cannot exceed 92 % [33]. So, the masking layer is designed to synergistically enhance solar reflectivity, especially in the ultraviolet band, to improve the full-band solar reflection. To match the peak reflection effect, we optimize the size of the TiO_2_ and SiO_2_ (Figures 3b and S1, Supplementary Material), TiO_2_ particle with a diameter of 500 nm can efficiently reflect visible light, while SiO_2_ particle with a diameter of 500 nm can mainly contribute to ultraviolet reflection. During scattering efficiency simulation, the background for TiO_2_ and SiO_2_ are resin and air, respectively.

The spectral reflectivity of the SBRC coating, TiO_2_/resin paint, and commercial paint are measured and presented in Figure 3c. The TiO_2_/resin paint has a high reflectivity above 90 % in the band from 410 nm to 1.3 μm that covers most visible and some near-infrared bands, and this wavelength region corresponds to the peak of solar radiation energy and accounts for more than 82 % of the total energy. On the other hand, an obvious ultraviolent absorption occurs for TiO_2_/resin paint, which weakened the total solar reflectivity to 89.2 %, but this value is close to the theoretical limit of the TiO_2_/resin paint. In addition, commercial paint shows similar solar reflectivity properties, but the total solar reflectivity is lower than that of TiO_2_/resin paint. This is because the main inorganic particle in commercial paint is also TiO_2_, but the size of the particle is not optimized. As for infrared optical properties, the TiO_2_/resin paint shows low reflectivity in the mid-infrared band with a weighted reflectivity of 4.6 %, corresponding to an emissivity of 95.4 %. The high emissivity of the TiO_2_/resin paint is mainly contributed by the large number of functional groups, such as C–H and C–O, contained in the acrylic polymer, and the vibration of these groups causes absorption of infrared light.

As described before, the superhydrophobic masking layer can improve the solar reflectivity of the TiO_2_/resin paint, and a significant reflectivity enhancement is observed in the ultraviolet with a reflectivity of over 85 %, which is contributed to the scattering effect of accumulation of SiO_2_ particles and intrinsic solar reflection of P(VdF-HFP) in the masking layer. Importantly, SiO_2_ and P(VdF-HFP) are nearly optically lossless, and the masking layer is very thin, so the masking layer has almost no negative impact on the high reflection of the coating in the remaining solar band. Based on the above mechanism, the AM1.5 weighted solar reflectivity of the SBRC coating reaches 94 %, exceeding the theoretical limitation of TiO_2_/acrylic coatings. In the infrared band, the high emissivity characteristic of SiO_2_ particle and P(VdF-HFP) results in a slight increase in the overall emissivity (97.1 %) in the mid-infrared band compared to the TiO_2_/resin paint, which indicates that the SBRC coating has potential for passive daytime radiative cooling.

### 3.3 Cooling performance and energy-saving potential

**Figure 4:**
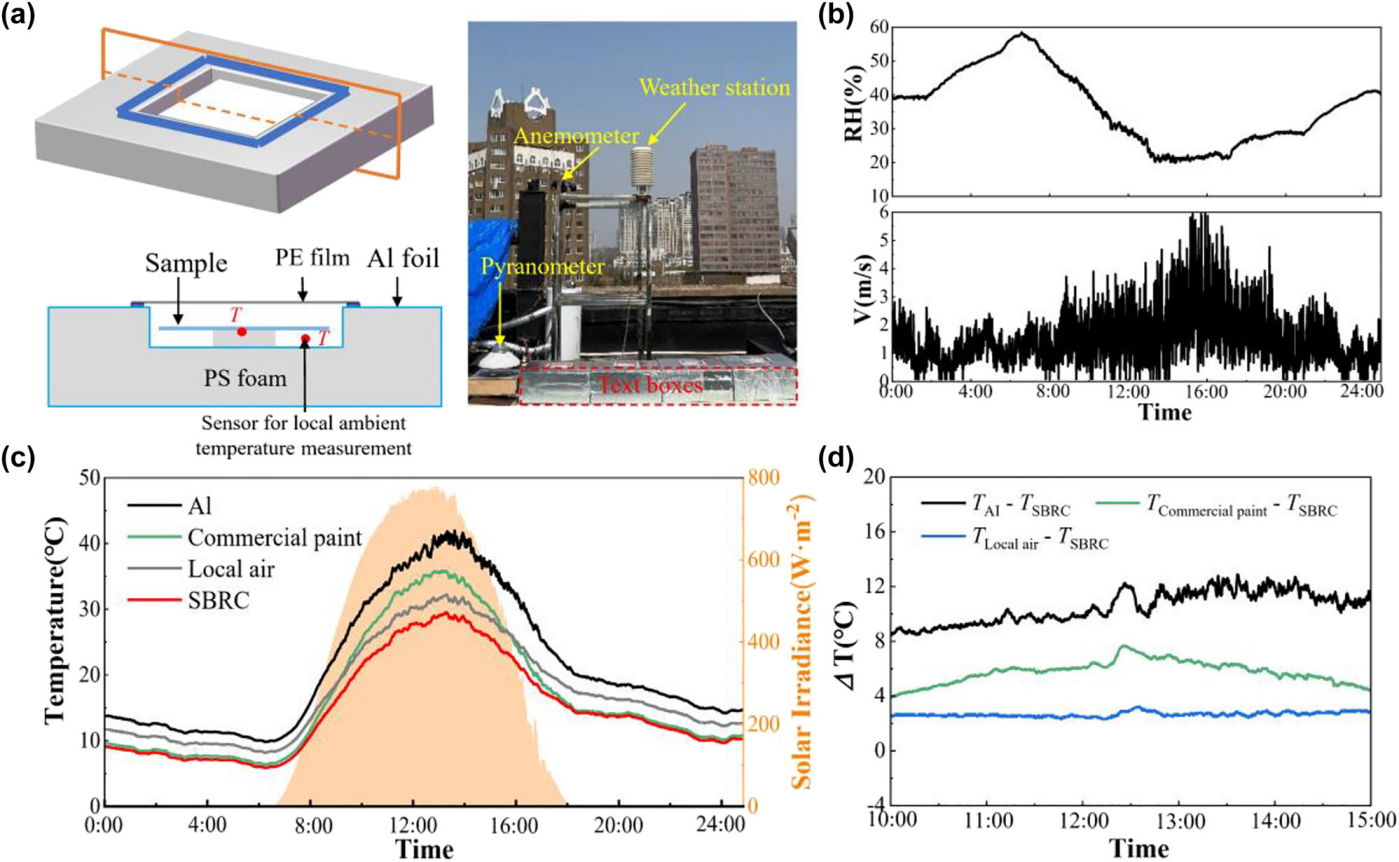
Experimental demonstration of radiative cooling with PE cover. (a) Schematic and photo of the test apparatus for the closed system. SBRC coating, Al, and commercial paint are fixed in the cavity with insulation supports and a PE cover. Al foil is used to cover the surface of the apparatus and thermocouples are used to measure the sample temperature and local ambient temperature. (b) Measured relative humidity (RH) and wind speed (V) during the testing period. (c) Measured stagnation temperatures of the SBRC coating, Al, and commercial paint, with local ambient temperature and solar irradiance plotted as references. (d) Temperature difference among SBRC and other samples at noon time from 10:00 to 15:00.

To test the radiative cooling performance of the SBRC, we design an outdoor experimental apparatus as illustrated in Figure 4a. SBRC coating, Al, and commercial paint are fixed in the cavity with insulation supports. The spectral property of the Al is shown in Figure S2 (see Supplementary Material). The stagnation temperature of the sample and the local ambient temperature underneath the sample are monitored. During the testing, relative humidity is below 60 % and the wind speed is at a relatively high level (Figure 4b). As described in Figure 4c, among all three samples, SBRC coating is always the coldest during the 24 h testing period, showing considerable radiative cooling performance. In addition, radiative cooling to below local ambient air under sunlight is only achieved by the SBRC coating, while that for commercial paint is destroyed by the solar heating effect. For the 5 h tested around noon, as shown in Figure 4d, the SBRC coating is 2.7 °C, 5.8 °C, and 10.5 °C cooler on average compared with local ambient air, commercial paint, and Al, respectively, even under solar irradiance of over 700 W m^−2^ on average. In addition, the maximal temperature reduction of the SBRC coating reaches 3.2 °C, 7.7 °C, and 12.9 °C compared with local ambient air, commercial paint, and Al, respectively. Compared with TiO_2_/resin paint, the SBRC coating can be further maximally cooler to 3.0 °C after introducing the masking layer for synergetic reflection (Figure S3, Supplementary Material).

**Figure 5:**
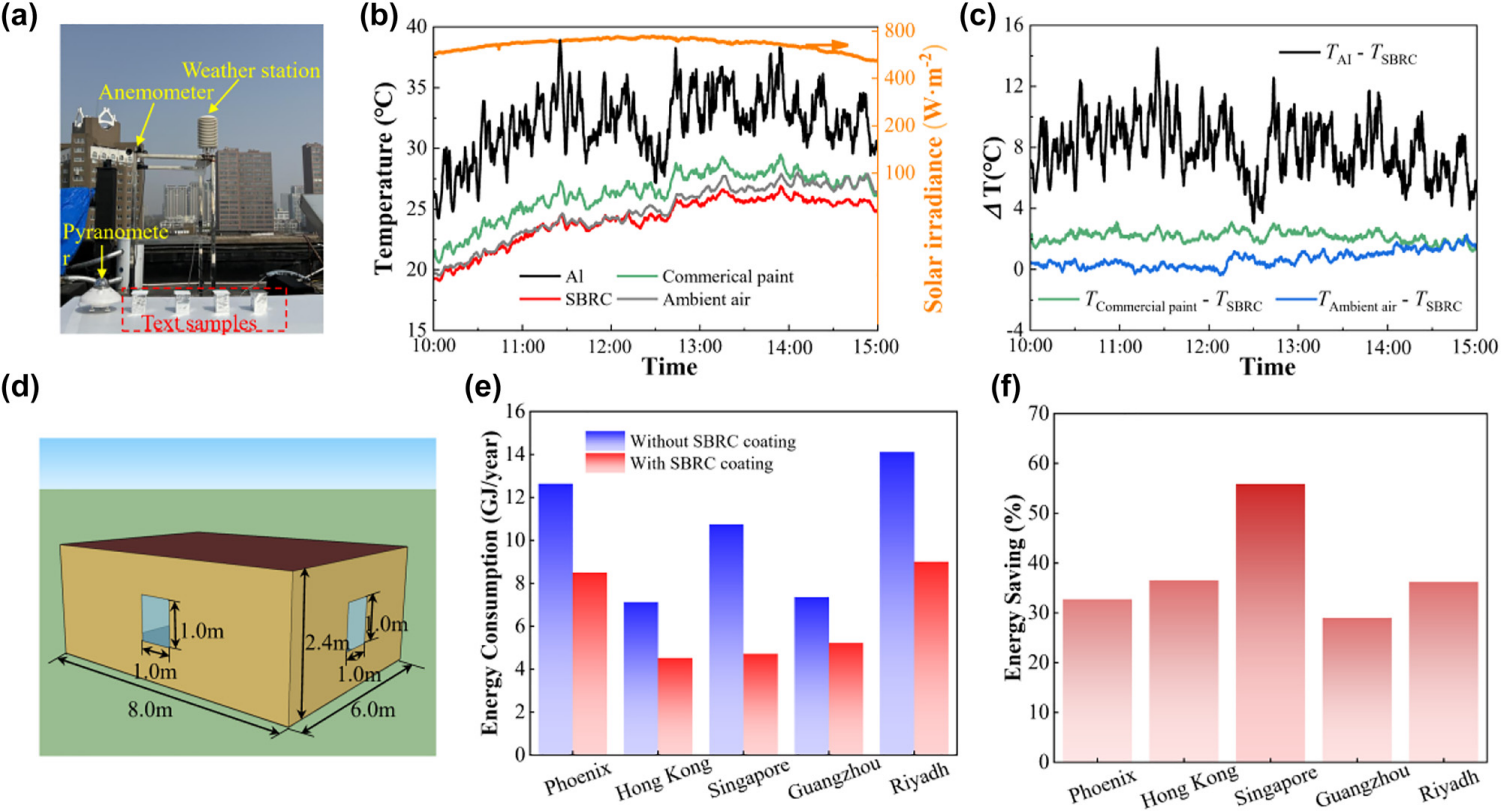
Radiative cooling of SBRC coating under the open system and energy saving potential of SBRC coating in buildings. (a) Photo of the test apparatus for the open system. SBRC coating, Al, and commercial paint are directly exposed to ambient air to stimulate real-world cases. (b) Measured stagnation temperatures of the SBRC coating, Al, and commercial paint, with ambient temperature and solar irradiance plotted as references. (c) Temperature difference among SBRC and other samples at noon time from 10:00 to 15:00. (d) Schematic of a building model for energy consumption evaluation. (e) Predicted yearly energy consumption of the building with and without SBRC coating in different cities. (f) Predicted energy saving of the SBRC coating in buildings in different cities.

To simulate real-world scenarios, an open system, as shown in Figure 5a, is set up for radiative cooling performance testing. SBRC coating, Al, and commercial paint are supported by thermally insulation pillars and directly exposed to the ambient air. The wind speed and relative humidity during the testing period are presented in Figure S4 (see Supplementary Material). As illustrated in Figure 5b and c, even at noon, the temperature of the SBRC coating is also the lowest compared with the commercial paint and Al with a temperature reduction of 2.1 °C and 8.0 °C, respectively, agreeing well with the results in the closed system. Notably, the temperature of the SBRC coating is almost the same as the ambient temperature, even at solar irradiance of 750 W m^−2^, showing considerable performance of heat dissipation. After 13:00, subambient cooling with a temperature reduction of 0.7 °C on average is achieved due to the degraded solar heating effect.

To further evaluate the energy-saving performance of the SBRC coating when integrated into buildings, we perform a large-scale building energy consumption simulation with and without SBRC coating in five different locations (Phoenix, Hong Kong, Singapore, Guangzhou, and Riyadh). A concise building (Figure 5d) is designed for prediction and the SBRC coating is assumed to cover the rooftop and exterior walls except windows with solar absorptivity/thermal emissivity of 6 %/97.1 % and thermal resistance of 0.02 m^2^ K W^−1^. In addition, the temperature setpoint for heating and cooling is 20 °C and 24 °C, respectively. The SBRC coating integrated building can save 4.13, 2.60, 6.00, 2.13, and 5.11 GJ/year at Phoenix, Hong Kong, Singapore, Guangzhou, and Riyadh, respectively, with an energy-saving percentage of 29.0 %–55.9 %.

## 4 Conclusions

In summary, a superhydrophobic bilayer radiative cooling (SBRC) coating is developed by introducing a SiO_2_/P(VdF-HFP) composite masking layer to the TiO_2_/acrylic resin paint, which can reflect 94.0 % incident sunlight and simultaneously exhibits a strong thermal emission with an average emissivity of 97.1 %. High visible reflection is attributed to the strong scattering effect of the TiO_2_/acrylic resin paint with optimized particle size. In addition, the SiO_2_/P(VdF-HFP) masking layer synergetically reflects ultraviolet light due to the scattering of the SiO_2_ particle and intrinsic reflection of the P(VdF-HFP). Importantly, the hydrophobically modified SiO_2_/P(VdF-HFP) masking layer provides a self-cleaning property for the SBRC coating with a water contact angle of 158.9°. Radiative cooling to 5.8 °C and 2.7 °C below commercial white paint and local ambient temperature under direct sunlight further is experimentally demonstrated. By integrating SBRC coating with the building, yearly energy saving of 29.0 %–55.9 % can be achieved in Phoenix, Hong Kong, Singapore, Guangzhou, and Riyadh. This work provides an alternative method to develop spectrum-tailored materials with large-scale fabrication and self-cleaning characteristics for radiative cooling, which may attract broad interest from the fields of renewable energy, material science, and energy-saving buildings.

## Supplementary Material

Supplementary Material Details
