# Supplementary material for: Superhydrophobic bilayer coating for passive daytime radiative cooling
Source: Nanophotonics. 2023 Oct 18;13(5):583–91. doi: 10.1515/nanoph-2023-0511 (PMC11501716; doi:10.1515/nanoph-2023-0511)
Supplement: Supplementary file 1 — Supplementary Material Details [file j_nanoph-2023-0511_suppl_001.docx]

**Supplementary Material for**

**Superhydrophobic bilayer coating for passive daytime radiative cooling**

Bin Zhao^1^, Chengfeng Xu^1^, Cheng Jin, Kegui Lu, Ken Chen, Xiansheng Li, Lanxin Li, and Gang Pei^*^

*Department of Thermal Science and Energy Engineering, University of Science and Technology of China, Hefei 230027, China*

* Corresponding author.

E-mail address: [peigang@ustc.edu.cn](mailto:peigang@ustc.edu.cn) (G. Pei)

^1^ These authors contributed equally to this work.

**Table S1** Information of envelope structures and thermophysical properties.

| Envelope | Material  (inside to outside) | Thickness  (m) | Thermal conductivity (W·m^-1^·K^-1^) | Density  (kg·m^-3^) | Specific heat  (J·kg^-1^·K^-1^) |
| --- | --- | --- | --- | --- | --- |
| Wall | Plasterboard | 0.012 | 0.16 | 950 | 840 |
|  | Fiberglass quilt | 0.066 | 0.04 | 12 | 840 |
|  | Wood siding | 0.009 | 0.14 | 530 | 900 |
| Floor | Concrete | 0.1015 | 1.7296 | 2243 | 837 |
| Roof | Plasterboard | 0.01 | 0.16 | 950 | 840 |
|  | Fiberglass quilt | 0.1118 | 0.04 | 12 | 840 |
|  | roof deck | 0.019 | 0.14 | 530 | 900 |
| Window | glass | 0.006 | 0.9 | - | - |
|  | air | 0.0032 | - | - | - |
|  | glass | 0.006 | 0.9 | - | - |


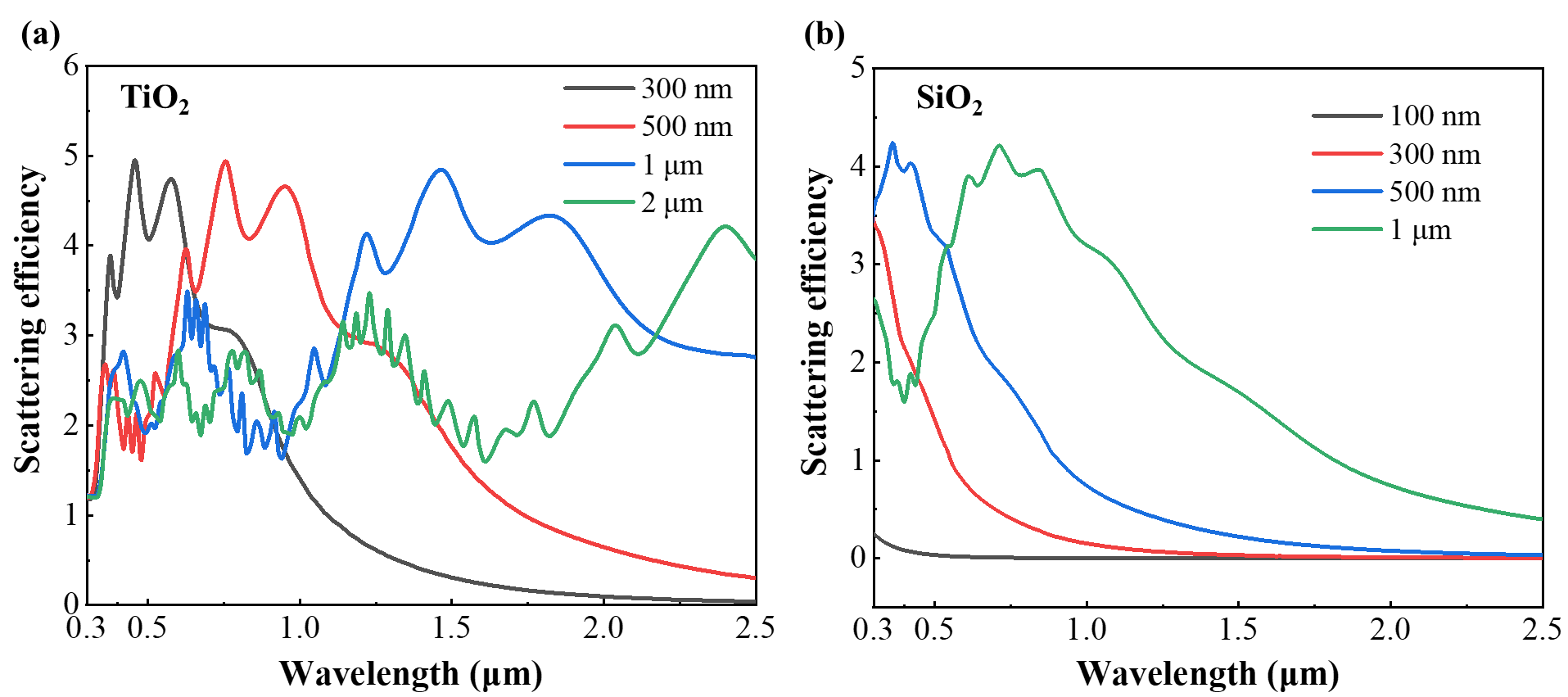


**Figure S1.** Scattering efficiency of (a) TiO_2_ and (b) SiO_2_ particles with different sizes.


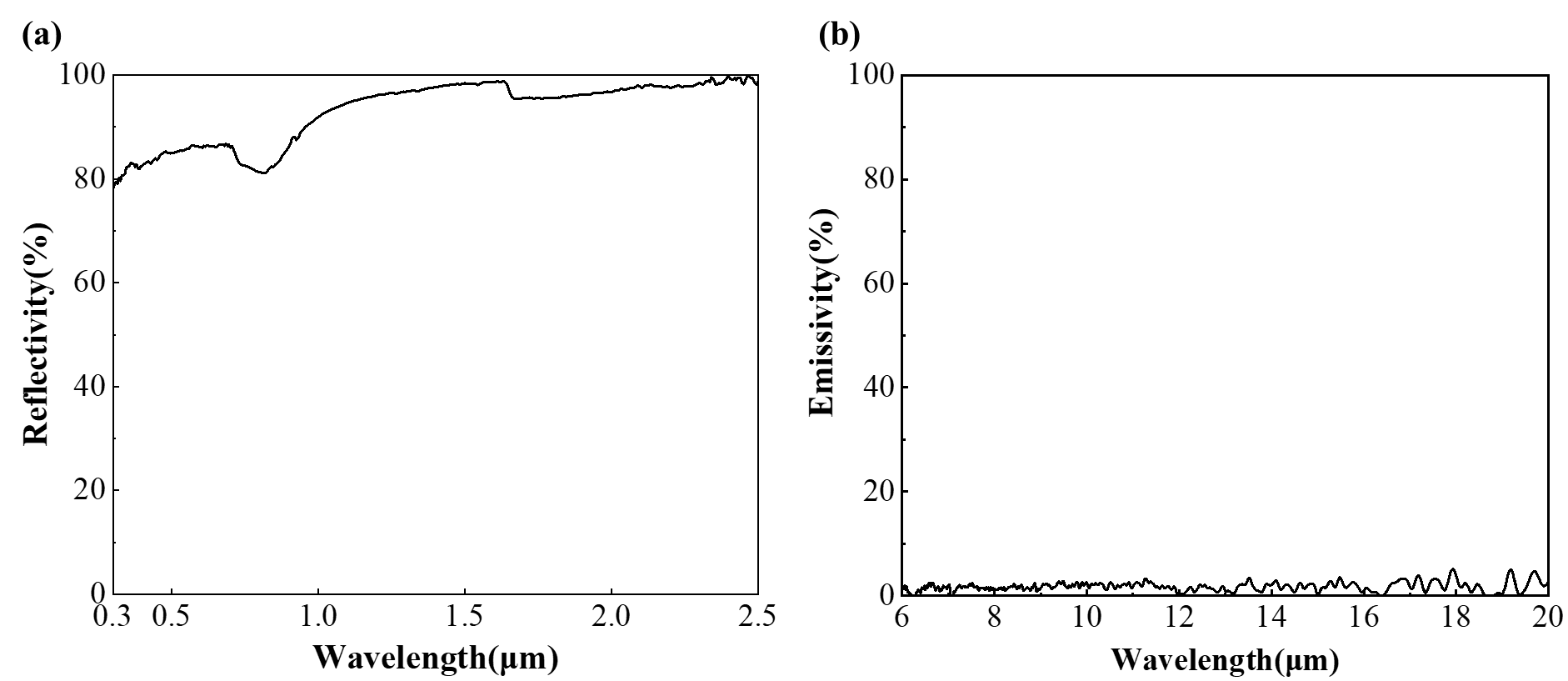


**Figure S2.** Measured reflectivity and emissivity of Al surface.

**Figure S3.** Stagnation temperature of the SBRC coating and TiO2/resin paint with solar irradiance plotted as a reference.


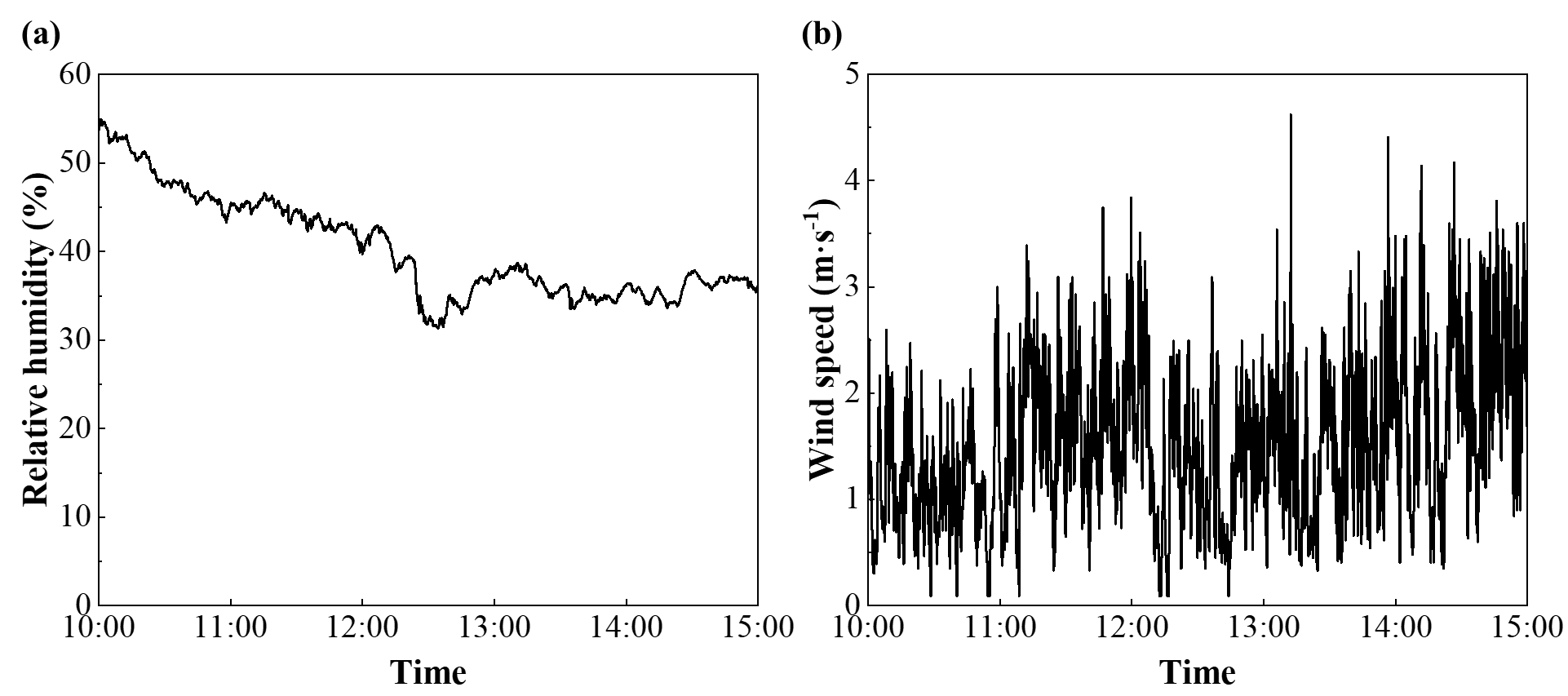


**Figure S4.** Measured relative humidity and wind speed during the testing of the open system.
